# Over-Population of the Towns

**Published:** 1888-09

**Authors:** 


### Over-Population of the Towns.

The rapid increase of the population accumulated in towns in this country, and the alleged growth of the class of unskilled laborers in towns engaged in trades which barely afford a livelihood, even when work is plentiful, has been engaging a good deal of attention lately; Prof. Huxley, and many other distinguished writers have taken part in the discussion without, however, contributing any solution. A "Committee for inquiry with the subject of over-population," has been formed, and will hold conference for the discussion of the subject on Mondays during May and June. Recent investigations have shown that there is a very much larger pauper immigration into this country than was generally supposed, and it is highly probable that some legislation to check this, similar to that already enforced in the United States, will be required. It would seem that a very large number of foreigners, chiefly, it is said, Polish Jews, have been coming over for many years past, and have gone into the tailoring and boot-making trades, with the result of very greatly depreciating wages. A select Committee of the House of Lords is at present taking evidence with regard to the "Sweating" system in the tailoring trade, and has elicited facts of a most harrowing nature; the House of Commons has also appointed a Select Committee on emigration and immigration, which has held several sittings.—*Pa. Medical Times.*

LONGEVITY IN JNAPAN.—Japan had, on January 1st, 1888, 38,507,177 people. Of these 1,085,001 were between 70 and 80 years old; 247,055 between 80 and 90; 12,220 between 90 and 100, and 97 over 100. Of these last there were 73 women and 24 men, two of the women being 109, and one 111 years old.

DR. MARY DURAND, director of the *Courrier Medical,* has been appointed a member of the Commission of Public Hygiene and Health of Paris.

